# Recovery of resting brain connectivity ensuing mild traumatic brain injury

**DOI:** 10.3389/fnhum.2015.00513

**Published:** 2015-09-22

**Authors:** Rose D. Bharath, Ashok Munivenkatappa, Suril Gohel, Rajanikant Panda, Jitender Saini, Jamuna Rajeswaran, Dhaval Shukla, Indira D. Bhagavatula, Bharat B. Biswal

**Affiliations:** ^1^Advanced Brain Imaging Facility, Cognitive Neuroscience Centre, National Institute of Mental Health and NeurosciencesBangalore, India; ^2^Department of Neuroimaging and Interventional Radiology, National Institute of Mental Health and NeurosciencesBangalore, India; ^3^Department of Clinical Neurosciences, National Institute of Mental Health and NeurosciencesBangalore, India; ^4^Department of Biomedical Engineering, New Jersey Institute of Technology, University HeightsNewark, NJ, USA; ^5^Neuropsychology Unit, Department of Clinical Psychology, National Institute of Mental Health and NeurosciencesBangalore, India; ^6^Department of Neurosurgery, National Institute of Mental Health and NeurosciencesBangalore, India

**Keywords:** mild traumatic brain injury, resting state functional connectivity, longitudinal study, time varying changes, brain plasticity, hyper connectivity

## Abstract

Brains reveal amplified plasticity as they recover from an injury. We aimed to define time dependent plasticity changes in patients recovering from mild traumatic brain injury (mTBI). Twenty-five subjects with mild head injury were longitudinally evaluated within 36 h, 3 and 6 months using resting state functional connectivity (RSFC). Region of interest (ROI) based connectivity differences over time within the patient group and in comparison with a healthy control group were analyzed at *p* < 0.005. We found 33 distinct ROI pairs that revealed significant changes in their connectivity strength with time. Within 3 months, the majority of the ROI pairs had decreased connectivity in mTBI population, which increased and became comparable to healthy controls at 6 months. Within this diffuse decreased connectivity in the first 3 months, there were also few regions with increased connections. This hyper connectivity involved the salience network and default mode network within 36 h, and lingual, inferior frontal and fronto-parietal networks at 3 months. Our findings in a fairly homogenous group of patients with mTBI evaluated during the 6 month window of recovery defines time varying brain connectivity changes as the brain recovers from an injury. A majority of these changes were seen in the frontal and parietal lobes between 3 and 6 months after injury. Hyper connectivity of several networks supported normal recovery in the first 6 months and it remains to be seen in future studies whether this can predict an early and efficient recovery of brain function.

## Introduction

Mild traumatic brain injury (mTBI) has an under reported incidence of 100–300 out of 100,000 cases and often gets little attention from the treating clinicians as it requires no emergent surgical treatment and has spontaneous recovery with predictable neurobehavioral sequelae (Cassidy et al., 2004). Around 5% of patients (Iverson, 2005) develop “post-concussion syndrome” (PCS) characterized by physical, emotional, cognitive, and sleep disturbances and may take many months to return to the baseline. Nevertheless, mTBI is considered a “silent epidemic,” given its incidence in economically active young men and so there is a priority in understanding the course of recovery and predictors for recovery in mTBI (Cassidy et al., 2004).

The predominant effort of neuroimaging in mTBI has been linked to brain measurements in neuropsychological assessments by comparing of patients with PCS at various time points post injury with healthy controls (Christodoulou et al., 2001; McAllister et al., 2001; Soeda et al., 2005; Maruishi et al., 2007; Sanchez-Carrion et al., 2008; Turner and Levine, 2008; Slobounov et al., 2010). Resting functional connectivity in mTBI found altered DMN (Hillary et al., 2011; Mayer et al., 2011; Johnson et al., 2012; Zhou et al., 2012), interhemispheric (Marquez de la Plata et al., 2011; Slobounov et al., 2011), motor (Kasahara et al., 2010), and executive network connectivity (Hillary et al., 2011; Mayer et al., 2011) in patients with PCS. Cross network ROI analysis has reported disruption between networks in the context of mTBI related memory deficits (Sours et al., 2013). Longitudinal studies have revealed persistent activation abnormalities in the dorsolateral prefrontal cortex in patients with PCS (Chen et al., 2008). Longitudinal DTI studies have shown (Eierud et al., 2014) increased fractional anisotropy in the acute phase reducing or normalizing in the chronic phase (Niogi and Mukherjee, 2010; Mayer et al., 2011; Munivenkatappa et al., 2014).

The neurometabolic cascade of TBI has been well-studied in animals, but the time frame for pathobiological events in rats is much shorter than for humans (Giza and Hovda, 2001). In humans, recovery has been documented by several longitudinal studies (Shanmukhi and Panigrahi, 2003; Sundström et al., 2004; de Boussard et al., 2005; Heitger et al., 2006; Tellier et al., 2009), which use neuropsychological tests to reveal that cognitive deficits could recover in a time period of 1 month to 1 year (Carroll et al., 2014). Since there are few published reports addressing normal recovery in mTBI using neuroimaging, we recruited acute mTBI patients within 36 h (R1) and longitudinally followed them at 3–4 months (R2) and 6–8 months (R3) months using clinical, neuropsychological and neuroimaging methods. We excluded potential confounds of PCS, by including only subjects who showed prominent neuro-cognitive recovery in 6 months for analysis. Our objectives were to investigate connectivity changes using resting state fMRI (rsfMRI) in a brain recovering from injury and to explore the relationship between resting functional connectivity and neurocognitive scores during recovery. Apart from comparing the functional connectivity between healthy controls and mTBI populations during different stages or recovery, we also performed within group comparisons to quantify normal recovery of mTBI subjects. The healthy control groups was only scanned once, as there were no significant changes in cognition and functional connectivity in a period of 6 months to 1 year (Damoiseaux et al., 2006; Beason-Held et al., 2009).

## Materials and methods

A total of 25 consecutive patients with mTBI and 21 healthy controls were recruited for this longitudinal study after NIMHANS (National Institute of Mental Health and Neuroscience, Bangalore, India) human ethics committee approval. All participants provided a written informed consent prior to the study. The operational definition of mTBI was kept uniform and included patients with histories of head traumas within 36 h of injuries with Glasgow Coma Scale (GCS) scores of 14–15, no abnormal findings on head CT scans, and satisfaction of at least one of the following criteria (1) Loss of Consciousness (LOC) lasting less than 30 min, (2) Post Traumatic Amnesia (PTA) lasting < 24 h, (3) Presence of any of the neurological symptoms like headaches, dizziness, seizures, and visual blurring. All patients were evaluated by experienced neurosurgeons (BID, DS) who had clinical experience in managing patients with brain injuries. The age, gender, and education-matched healthy volunteers were recruited among the students and working staff. Exclusion criteria for both patients and controls were neuropsychiatric illness/symptoms, neuroinfection, past neurological/surgical diseases, intoxication, alcoholic or drug dependence, past history of head injury, left handedness, any abnormal findings in anatomical MRI, current or pending litigation and any medication which could alter the analysis. Post-concussion symptoms were assessed using the Rivermead post-concussion symptom questionnaire (RPQ). We excluded patients with presence of four or more concussion symptoms (Rivermead concussion scale sub scores more than 2) at 6 months for the final analysis.

### Neuropsychological tests

The tests were selected from the neurotrauma battery of NIMHANS neuropsychology battery of cognitive tests standardized for Indian populations (Rao et al., 2004). The neurotrauma battery includes the Digit symbol substitution test (DSST), Digit vigilance test (DVT) for attention, Animal naming test (ANT), Spatial span test, Verbal n back test (VnB), and Stroop test for testing executive functions. Learning and memory were assessed using Rey's auditory verbal learning test (RAVLT) and Rey's complex figure test (CFT). These neuropsychological assessments were performed at R1, R2, and R3. The details of the scores at R1, R2, and R3 are provided in Table 1.

**Table 1:** **Comparison of neuropsychological test scores that were assessed with serial intervals**.

	**R1**	**R2**	**R3**	***F*-test**	***p-*Values**
Animal naming test	12.82±2.69	13.12±3.08	14.35±2.6	7.63	0.002^**^
Digit symbol substitution test	236.18±102.9	207.47±88.04	203.12±84.3	8.58	0.008^**^
Digit vigilance test	486.65±115.5	451.41±131.18	422.24±116.17	10.57	<0.001^*^
Stroop test	116.71±45.8	93.35±34.4	87.65±34.28	13.74	0.001^**^
Spatial span	13.41±2.8	14.76±2.3	15.35±2.6	6.1	0.016^**^
Verbal n back 1	8.29±1.04	8.82±0.39	8.94±0.243	6.48	0.016^**^
Verbal n back 1 errors	0.82±1.074	0.18±0.39	0.06±0.24	8.46	0.007^**^
Verbal n back 2	6.88±1.45	7.88±0.85	8.41±0.71	16.28	<0.001^**^
Verbal n back 2 errors	2.41±1.5	0.59±0.71	1.41±1.0	23.09	<0.001^**^
Complex figure test Copy	35.21±1.33	35.91±0.26	36±0	5.36	0.032^**^
Complex figure test IR	21.38±7.99	25.83±6.08	27.85±4.96	12.57	0.001^**^
Complex figure test DR	22.03±7.94	25.74±6.6	28.06±6.95	7.32	0.01^**^
Auditory verbal learning test T	51.35±11.71	53.41±12.65	60.71±8.99	13.23	<0.001^*^
Auditory verbal learning test IR	10.24±3.38	11±3.2	11.82±2.6	3.5	0.066^*^
Auditory verbal learning test DR	10.35±3.44	10.65±3.9	11.71±2.99	3.07	0.78^**^
Auditory verbal learning test H	14±1.58	14.06±2.3	14.53±1.28	0.638	0.49^**^
Auditory verbal learning test LTPR	83.15±16.53	82.34±24.78	85.01±13.67	0.24	0.78^*^

*E, early assessment (2–3 weeks mean of 12.71 days); S, second assessment (3 months mean of 105.88 days); T, third assessment (6 months mean of 226.18 days); IR, immediate recall; DR. delayed recall; T, total count; H, total hits; LTPR, long term potential retention*.

*p-Value is computed by repeated measure of analysis; Significant p ≤ 0.05 is denoted in bold*.

If p-value has

** Greehouse–Geisser test and if

* *sphericity assumed*.

### Image acquisition

During the data acquisition, subjects were instructed to remain in a relaxed state without engaging in cognitive or motor activity and to keep their eyes closed. Resting Functional MR-images were acquired using a 3T scanner (Skyra, Siemens, Erlangen, Germany). One hundred and eighty-five volumes of Spin echo Echo-Planar Images (EPI) were obtained using the following EPI parameters: 36 slices, 4 mm slice thickness in interleaved manner with an FOV of 192 × 192 mm, matrix 64 × 64, repetition time 3000 ms, echo time 35 ms, flip angle 90°, voxel size 3 × 3 × 4 mm. We also acquired 3D MPRAGE (three dimensional magnetization-prepared rapid acquisition gradient echo) sequence for anatomical information (with the voxel size 1 × 1 × 1 mm, 192 × 256 matrix) for better registration and overlay of brain activity. The number of slices was 34 (slice thickness 3.75 mm) for the rsfMRI sequences at R2 and R3. Other parameters remained unchanged on follow up.

### Image analysis

For each subject, data processing scheme based on SPM8 software (SPM8; Welcome Department of Cognitive Neurology, London) was implemented as defined in our previous paper (Bharath et al., 2015a). In the first step, the first five functional images were discarded from each of the subjects' rsfMRI data to account for T1 relaxation effects. After discarding the first five images, each of the remaining images was registered to mean image to correct for head movements within the scan using the “realign” tool in SPM8. During the motion correction step, head motions in six directions (x, y, z, roll, pitch, and yaw) were derived. Following realignment, each of the functional images were co-registered to anatomical MPRAGE images for each subject. Next, MPRAGE images were segmented into gray matter, white matter (WM), and cerebral spinal fluid (CSF), and distinct probability maps were derived for each of these segments, using the “new segment” tool in SPM8. During segmentation, deformation fields were calculated to transform the MPRAGE images into MNI (Montreal Neurologic Institute) standard space. For the subjects, WM/CSF probability maps were thresholded at *p*>0.99 to derive binary masks representing WM/CSF. Each of the subjects' functional image was transformed in MNI standard space using the deformation field derived in the new segmentation procedure and down sampled to 3 mm isotropic voxels to make group comparisons feasible. The subjects' WM/CSF masks were used to extract time series from EPI data pertaining to WM and CSF. These time-series were extracted using in-house developed MATLAB scripts that used built-in SPM^*^ functions (spm_read_vol.m). Principal component analyses were performed using the “PRINCOMP” function in MATLAB(R2012b). A total of 34 nuisance time-series were used as covariates in linear regression models to minimize the effects of physiological and motion signals. These included the first five principal components of WM and CSF time-series, six time series describing head motion, six time-series describing head motion at previous time points and 12 quadratics of motion time-series (Friston 24-model). Following linear regression, each of the subjects BOLD fMRI data was spatially smoothed with 6 mm FWHM Gaussian blur and temporally filtered between 0.01 and 0.1 Hz. For all the further analysis, we used the temporally filtered data between 0.01 and 0.1 Hz henceforth reported as processed rsfMRI data.

### Connectivity analysis

In order to study whole brain connectivity analysis, we implemented group level independent component analysis using the MELODIC software in FSL (FMRIB's Software Library, www.fmrib.ox.ac.uk/fsl). Group ICA analysis was performed using temporal concatenation approach by combining processed rsfMRI data from both the healthy controls and mTBI subjects across the three runs. A total of 20 IC maps were derived using the. Each of the IC maps was compared with the Group ICA maps derived in FCP-1000 project to identify resting state networks using AFNI tools (Taylor and Saad, 2013). For each of the ICs that correspond to the known RSNs, we divided the IC maps into spatially independent clusters and peak coordinates were derived for each of the clusters. A 6 mm sphere were created around this peak coordinates to create a spherical ROIs in the MNI standard space. For each of the ROIs, we extracted a mean BOLD time series from each of the subjects' processed rsfMRI data (following regression and temporal band-pass filtering). We calculated Pearson's correlation coefficients between mean time series for each of the ROI pairs as a measure of resting state functional connectivity (RSFC) strength. These correlation coefficients were later transformed into Fishers-Z scores. Group level two-sample *t*-test was performed to compare RSFC strength between controls subjects and mild TBI population at different recovery periods. In order to identify an overall change in RSFC strength of the mTBI group, we first performed a One-Way ANOVA using time as a factor to derive changes in RSFC strength as a whole. Large-scale changes in RSFC strength were observed in mTBI population (*p* < 0.05, results not shown). In order to further classify functional connectivity changes in the mTBI population during recovery periods and follow a similar approach to compare the functional connectivity differences between controls and mTBI population, we also performed paired *t*-test between each of the TBI runs to quantify the recovery paradigm. For all the group level comparisons (HC vs. mTBI at various time points and within mTBI group comparisons), the significance level was kept the same at *p* < 0.005. Each of the ROI pairs showing significant group level differences were visualized on the brain surface using the BrainNet viewer (http://www.nitrc.org/projects/bnv/).

### Behavior correlation

An exploratory analysis was performed to drive the relationship between recovery of mTBI population quantified using RSFC strength and the recovery of mTBI subjects defined using neurocognitive tests. For each of the ROI pairs that show group level differences between the ROI pairs, we extracted the Pearson's correlation coefficient for each of the subject. This correlation coefficient representing the RSFC strength was than transformed in the Fishers *Z*-scores. In order to derive relationship between changes in RSFC strength and corresponding changes in neurocognitive scores, we calculated Pearson's correlation coefficient between each of the ROI pairs and corresponding neurocognitive scores. Significance level for this exploratory analysis was kept at *p* < 0.01 to derive the significant correlation between ROI pairs and behavior correlations.

## Results

Because head motion is a concern in most fMRI studies, especially resting state studies (Stamelou et al., 2012; Van Dijk et al., 2012), all the data from each of the subjects was tested for the presence of excessive head motion. Motion correction was performed using SPM8. During the motion analysis, two subjects from mTBI group and one subject from HC were excluded because they had more than 0.15 mm (maximum frame wise displacement) movement during the study.

A total of eight mTBI subjects who had concussion symptoms of more than four with a RPQ sub score more than 2 at 6 months were excluded from the study, as the aim of our study was to visualize normal recovery pattern. For further analysis, we had 15 subjects with mild TBI evaluated thrice and 20 HC. There were no significant differences in the demographic variables between the mTBI patients and HC. Road traffic accidents (RTA) 16(61.5%) were major cause of injuries followed by falls 7(26.9%) and assault 3(11.5%). The mean GCS score was 14.96, standard deviation was 0.2, and range was 14–15. All patients had LOC with a mean duration of 11.24 min, standard deviation of 10.19 (min), and range 1–30 (min). Imaging studies were performed on the same day as behavioral assessment, on follow-up. As the patients could not be attentive to the tasks at the time of R1, the first behavioral assessment was performed as soon as the patients were comfortable. Mean duration of injury to R1 was 19.00 h (standard deviation 11.33, range 3–36) and to the first neuropsychological assessment was 12.71 days. Mean duration of injury to R2 and the second neuropsychological assessment was 105.88 days; R3 and third neuropsychological assessment was 226.18 days. None of the patients were on regular medications like anti-depressants or anxiolytics during follow ups. Common symptoms present during follow ups were headaches, fatigue, sleep disturbances, irritability, poor concentration etc.

### Functional connectivity differences

#### Independent component analysis maps

**Figure 1:**
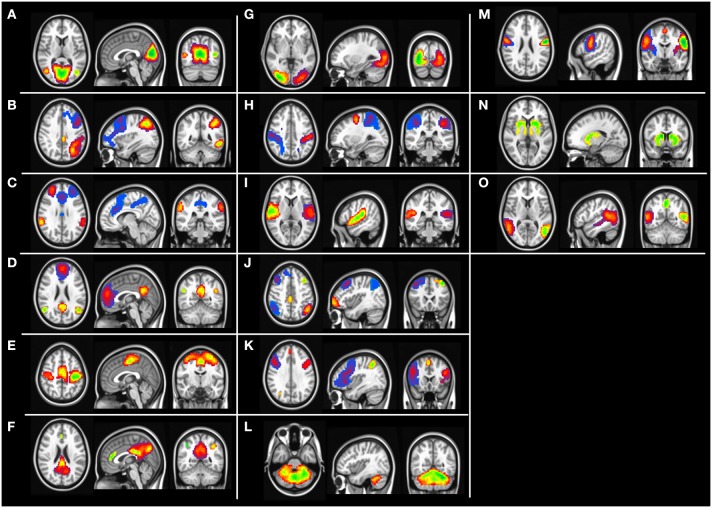
**Group level independent component maps**. **(A)** Lingual gyrus, **(B)** left frontal parietal network, **(C)** Salience network, **(D)** default mode network, **(E)** motor network, **(F)** posterior default mode network, **(G)** higher visual cortex, **(H)** Dorsal attention network, **(I)** Inferior frontal gyrus, **(J)** Fronta-parietal network, **(K)** Insular network, **(L)** cerebellum network, **(M)** pre-central gyrus, **(N)** basal ganglia, **(O)** temporal gyrus network.

Group level independent component analysis revealed 15 visually identifiable resting state networks (RSN) as demonstrated in Figure 1. For each of the identified RSN, we segmented the IC maps into non-contiguous clusters and peak voxel-coordinates were derived using AFNI program 3dClustSim. A 6 mm sphere was created surrounding the peak coordinate to derive a set of 57 ROIs that are listed in the Table 2.

**Table 2:** **The ROI with their coordinates created from the 15 derived networks**.

**IC number**	**ROI number**	**ROI number**	***X***	***Y***	***Z***	**ROI name**
IC01, lingual gyrus	ROI01	ROI01	−3	73	13	Right_Cuneus
	ROI02	ROI02	−45	70	10	Right_Middle_Temporal_Gyrus
	ROI03	ROI03	42	73	13	Left_Middle_Temporal_Gyrus
IC02, L fronto−parietal	ROI01	ROI04	30	64	43	Left_Superior_Parietal_Lobule
	ROI02	ROI05	45	−32	22	Left_Middle_Frontal_Gyrus
IC03, salience network	ROI01	ROI06	−30	−38	34	Right_Middle_Frontal_Gyrus
	ROI02	ROI07	3	−29	31	Left_Cingulate_Gyrus
	ROI03	ROI08	33	−35	37	Left_Middle_Frontal_Gyrus
	ROI04	ROI09	54	46	37	Left_Supramarginal_Gyrus
	ROI05	ROI10	−60	40	31	Right_Supramarginal_Gyrus
	ROI06	ROI11	39	−14	4	Left_Insula
	ROI07	ROI12	18	−8	64	Left_Superior_Frontal_Gyrus
	ROI09	ROI13	−18	−11	61	Right_Middle_Frontal_Gyrus
	ROI10	ROI14	−42	−17	1	Right_Insula
IC04, fronto−parietal network	ROI01	ROI15	0	−50	19	Left_Medial_Frontal_Gyrus
	ROI02	ROI16	6	52	28	Left_Cingulate_Gyrus
	ROI03	ROI17	48	64	31	Left_Angular_Gyrus
	ROI04	ROI18	−48	61	31	Right_Angular_Gyrus
IC05, motor network	ROI01	ROI19	33	25	64	Left_Precentral_Gyrus
	ROI02	ROI20	−33	25	64	Right_Precentral_Gyrus
	ROI03	ROI21	3	19	55	Left_Medial_Frontal_Gyrus
IC06, posterior default mode network	ROI01	ROI22	9	67	34	Left_Precuneus
	ROI02	ROI23	39	55	43	Left_Inferior_Parietal_Lobule
	ROI03	ROI24	0	−41	10	Right_Anterior_Cingulate
	ROI04	ROI25	−39	55	46	Right_Inferior_Parietal_Lobule
IC07, higher visual cortex	ROI01	ROI26	−33	85	4	Right_Middle_Occipital_Gyrus
	ROI02	ROI27	27	88	7	Left_Middle_Occipital_Gyrus
IC08, default mode network	ROI01	ROI28	36	40	49	Left_Inferior_Parietal_Lobule
	ROI02	ROI29	−36	40	49	Right_Inferior_Parietal_Lobule
	ROI03	ROI30	27	4	52	Left_Middle_Frontal_Gyrus
	ROI04	ROI31	−27	4	52	Right_Middle_Frontal_Gyrus
	ROI05	ROI32	54	−8	25	Left_Inferior_Frontal_Gyrus
	ROI06	ROI33	−54	−8	25	Right_Inferior_Frontal_Gyrus
	ROI07	ROI34	57	22	40	Left_Postcentral_Gyrus
	ROI08	ROI35	−57	22	40	Right_Postcentral_Gyrus
	ROI09	ROI36	−51	52	−8	Right_Middle_Temporal_Gyrus
	ROI10	ROI37	48	61	−5	Left_Middle_Occipital_Gyrus
IC09, dorsal attention network	ROI01	ROI38	−60	22	16	Right_Postcentral_Gyrus
	ROI02	ROI39	60	22	13	Left_Superior_Temporal_Gyrus
	ROI03	ROI40	0	−47	−8	Left_Medial_Frontal_Gyrus
IC10, inferior frontal	ROI01	ROI41	39	−56	−5	Left_Middle_Frontal_Gyrus
	ROI02	ROI42	−39	−56	−5	Right_Middle_Frontal_Gyrus
	ROI03	ROI43	63	31	−11	Left_Middle_Temporal_Gyrus
	ROI04	ROI44	−63	22	−14	Right_Middle_Temporal_Gyrus
	ROI05	ROI45	−3	28	37	Right_Cingulate_Gyrus
IC12, cerebellum network	ROI01	ROI46	27	58	−26	Left_Culmen
	ROI02	ROI47	−27	58	−26	Right_Culmen
IC13, insular network	ROI01	ROI48	−48	−20	28	Right_Middle_Frontal_Gyrus
	ROI02	ROI49	45	−20	25	Left_Middle_Frontal_Gyrus
	ROI03	ROI50	−3	−29	49	Right_Superior_Frontal_Gyrus
IC15, pre−central gyrus	ROI01	ROI51	57	4	25	Left_Precentral_Gyrus
	ROI02	ROI52	−57	4	28	Right_Precentral_Gyrus
	ROI03	ROI53	0	1	58	Left_Medial_Frontal_Gyrus
IC17, basal ganglia	ROI01	ROI54	12	−2	10	Left_Caudate
	ROI02	ROI55	−15	−5	13	Right_Caudate
IC18, temporal gyrus	ROI01	ROI56	57	46	16	Left_Superior_Temporal_Gyrus
	ROI02	ROI57	−57	46	16	Right_Superior_Temporal_Gyrus

#### Whole brain mean connectivity analysis

**Figure 2:**
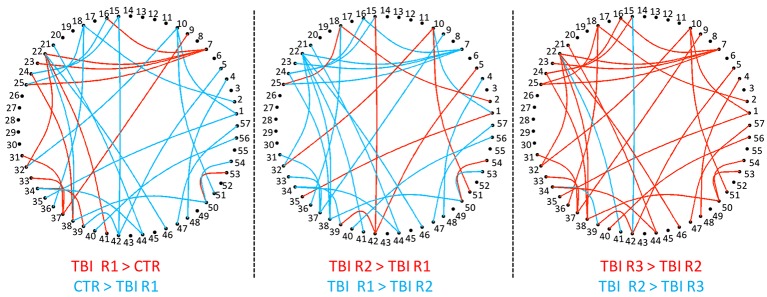
**Group level RSFC differences during recovery from mTBI within 36 h (Control > R1), at 3 months (R2 > R1), and at 6 months (R3 > R2)**. The decreased connectivity is depicted as edges. The nodes, which are numbered, depicts the ROI seed pairs. Progressively increasing connectivity shown as red edges is most evident at 6 months.

Group level differences in whole brain RSFC revealed 33 ROI pairs that showed significant mean connectivity differences existing based on group level comparisons between healthy controls and mTBI patients and within mTBI patients (Table 3). Both the TBI group analysis and the comparison with control group analysis revealed that major functional connectivity changes occurred in brain between 3 and 6 months after injuries. Most of the ROI pairs revealed decreased connectivity in mTBI subjects in the first 3 months (marked blue in Figure 2) and increased connectivity between 3 and 6 months (marked red in Figure 2). These connectivity differences were diffused and bilateral but prominently involved the frontal and parietal lobes.

**Table 3:** **RSFC ROI pairs that revealed significant differences with time with their abbreviations used in the figures and text**.

**Serial number**	**Abbreviations used**	**Names of the RSFC ROI pairs**
1	R-SFG–L-caudate	Right Superior Frontal Gyrus to Left Caudate
2	R-SFG–R-MIG	R-Superior Frontal Gyrus–R Medial Frontal Gyrus
3	R-Culmen–R STG	R-Culmen–R Superior temporal Gyrus
4	L-MIG–L STG	R-Medial Frontal Gyrus–L Superior temporal Gyrus
5	L-STG–R-MFG	L-superior temporal gyrus–R-Middle Frontal Gyrus
6	R-POG–R-SFG	R-postcentralgyrus–R-Superior Frontal gyrus
7	L-POG–R-MTG	L-post central gyrus–R-Middle Temporal Gyrus
8	L-POG–L-STG	L-Post Central Gyrus–L-Superior Temporal Gyrus
9	R-IFG–L-MOG	R-Inferior Frontal Gyrus–L-Middle Occipital Gyrus
10	R-MFG–L-MOG	R-Middle Frontal Gyrus–L-middle Occipital Gyrus
11	R-ACC–R-POG	R-Anterior Cingulate Cortex–R-Post Central Gyrus
12	L-precuneus–L-culmen	L-Precuneus–L-Culmen
13	L-Precuneus–L-MFG	R-Precuneus–L-Middle Frontal Gyrus
14	L-precuneus–R-POG	L-Precuneus–R-Post Central Gyrus
15	L-Precuneus–L-IFG	L-Precuneus–L-Inferior Frontal Gyrus
16	L-MFG–R-MTG	L-Middle Frontal Gyrus–R-Middle Temporal Gyrus
17	R-AngGyrus–R-MTG	R-Angular Gyrus–R-Middle Temporal gyrus
18	R-AngGyrus–R-IPL	R-Angular Gyrus–R-Inferior Parietal Lobule
19	L-CG–RACC	R-Cingulate Gyrus–R Anterior Cingulate Cortex
20	L-MIG–R-MFG	L-Medial Frontal Gyrus–R-Middle Frontal Gyrus
21	L-MIG–R-ACC	L-Medial Frontal Gyrus–R-Anterior Cingulate Cortex
22	R-SMG–L-PCG	R-SupramarginalGyrus–R-precentralgyrus
23	R-SMG–L-IFG	R-SupramarginalGyrus–L-Inferior Frontal Gyrus
24	L-SMG–L-MOG	L-SupramarginalGyrus–L-Middle Occipital Gyrus
25	L-CG–R-IPL	L-CingualteGyrus–R-inferior Parietal Lobule
26	L-CG–L-IPL	L-CingualteGyrus–L-Inferior Parietal Lobule
27	L-CG–L-Precuneus	L-CingualteGyrus–L-Precuneus
28	L-CG–L-CG	L-Cingulate Gyrus–L-Cingulate Gyrus
29	L-MFG–R-MFG	L-Middle Frontal Gyrus–R-Middle Frontal Gyrus
30	L-SPL–R-MFG	L-Superior Parietal lobule–R-Middle Frontal Gyrus
31	R-MTG–R-AngGyrus	R-Middle Temporal Gyrus–R-Angular gyrus
32	R-Cuneus–R-POG	R-Cuneus–R-Post Central Gyrus
33	R-cuneus–R-SMG	R-Cunues–R-supramarginalGyrus

*Serial numbers 1–15 are the pairs that were significantly different in the within subject group analysis. Serial numbers 16–33 are the ROI pairs that were different in comparison with the healthy controls*.

#### RSFC differences as function of recovery within mTBI patients (R1 vs. R2 vs. R3)

**Figure 3:**
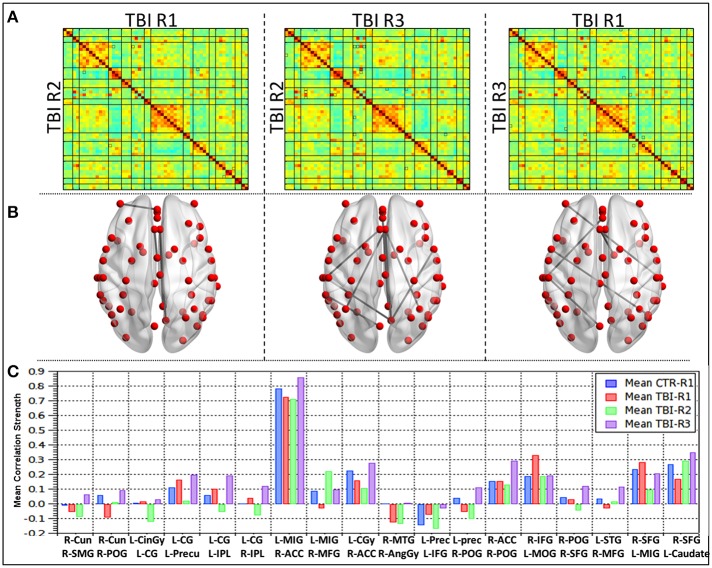
**Group level differences observed in the RSFC within mild TBI subjects during recovery**. **(A)** Mean correlation matrices for each of the groups, **(B)** ROI pairs showing differences in the connectivity within mild TBI (*p* < 0.005), **(C)** mean connectivity strength for each of the ROI pair showing differences during recovery in mild TBI subjects.

Comparison of RSFC strength within mTBI group (Figure 3) revealed 18 ROI pairs that showed significant connectivity differences during the recovery period. Between 36 h and 3 months (R1 vs. R2) only a few ROI pairs revealed significant connectivity differences. Most of the ROI pairs revealing significant differences were evident in the contrasts linked to R3 (R2 vs. R3 and R1 vs. R3). Areas that showed significant changes in connectivity were clustered within the bilateral parietal lobes (right post central gyrus, right angular gyrus, right supramarginal gyrus, bilateral inferior parietal lobule, left precuneus), bilateral frontal lobes (right superior middle and bilateral inferior frontal gyrus, left medial frontal gyrus), and midline structures anterior cingulate, left cingulate gyrus. Significant differences were also observed in right middle temporal gyrus, right cuneus, and left middle occipital gyrus.

A detailed analysis of RSFC strength for each of the 18 ROI pairs (Figure 3C) revealed that comparisons between R1 (acute phase) and healthy controls 10 ROI pairs (56%) revealed decreased connectivity and seven ROI pairs (39%) had increased connectivity. One ROI pair (L-SPL–R-MFG) had no significant change. The networks that revealed increased connections prominently involved the salience network, DMN, and Posterior DMN as well as insular, precentral, and fronto-parietal network. The ROI pairs, which revealed increased connections, were predominantly left hemispheric and involved the connections of the left cingulate gyrus within itself and to the bilateral IPL and left precuneus. R IFG–L MOG, L IFG–left precuneus, and L MIG–R SFG also showed increased connectivity. Decreased connectivity was predominantly right sided and involved right parietal lobes (R AG–R MTG, R SMG–R Cun, R POG-R Cun, R POG–R ACC, R POG–R SFG, R POG–L precun), right frontal lobes (R MFG-L MiFG, R MFG-L STG, R SFG–L Caudate), and ACC (R ACC-L MiFG, R ACC-L CG). Subsequent comparisons between mTBI subjects between R2 and R1, revealed that a majority of the network and 70% ROI pairs had decreased connections. Salience network, anterior DMN, posterior section of DMN and the ROI pairs which showed increased connections at R1 revealed decreased connections at R2. One ROI pair, R-POG–R-ACC remained unchanged between R1 and R2. Increased connections of lingual-DMN, inferior frontal networks to fronto-parietal and dorsal attention networks and insular to basal ganglia networks were also observed. All the four ROI pairs that showed increased connections in R2 had decreased connections at R1 and persistently showed increased connectivity at R3. All four except one (R POG-R Cun) were interhemispheric and involved R MFG-L MiFG, R MFG-L STG, and R SFG–L Caudate. In the chronic phase (R2 vs. R3), all the ROI pairs except one (89%) had increased RSFC strength. The RSFC strength between R-MFG and L-MiFG decreased from 3 to 6 months period.

#### RSFC differences of mTBI patients in comparison with healthy control

**Figure 4:**
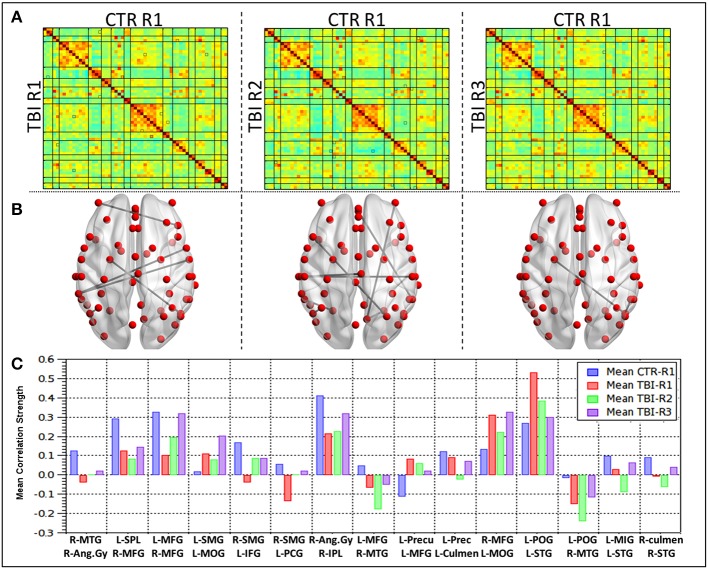
**Group level differences observed in the RSFC between healthy controls and TBI subjects during recovery**. **(A)** Mean correlation matrices for each of the groups, **(B)** ROI pairs showing differences in the connectivity between HC and mild TBI (*p* < 0.005), **(C)** mean connectivity strength for each of the ROI pair showing differences between the HC and mTBI subjects.

Group level comparisons with healthy controls (Figure 4) revealed 15 ROI pairs, which had significant connectivity differences during the recovery period. No overlap was observed between within mTBI group differences and the group comparison between controls and mTBI subjects. Large-scale differences in RSFC strength were observed in the contrast between HC vs. R1 and HC vs. R2 i.e., patients were different from healthy control at R1 and R2. The functional connectivity differences at R3, however, were minimal. This finding is in agreement with the results of the mTBI group analysis mentioned above. Significant changes in connectivity were seen within the bilateral parietal lobes (right post central gyrus, right angular gyrus, right supramarginal gyrus, left superior parietal lobule, left precuneus) and bilateral frontal lobes (right superior middle and bilateral inferior frontal gyrus, left medial frontal gyrus). Involvement of other areas like the right middle temporal gyrus, left superior temporal gyrus, bilateral culmen, and left middle occipital gyrus were also observed.

Detailed analysis of mean connectivity strength of 15 ROI pairs (Figure 4C) revealed that at R1 (Acute phase) 11 seed pairs (73%) revealed decreased connectivity and four ROI pairs (23%) had increased connectivity in comparison with the healthy controls. The networks revealing increased connections prominently involved the salience network, DMN, Posterior DMN network apart from inferior frontal and dorsal attention networks. The ROI pairs that revealed increased connections predominantly involved the left middle occipital gyrus (L MOG–L SMG, L MOG-R MFG) and left parietal lobe (L POG–L STG, L Precun–L MFG). The connections that revealed a decrease prominently involved bilateral parietal lobes (R Ang Gyr-R MTG, R Ang Gyru–R IPL, R SMG–L IFG, R SMG-L PCG, L POG–R MTG, L SPL–R MFG, L Precun–L culmen) and the left frontal lobe (L MFG–R MFG, L MFG–R MTG, L MIG–L STG apart from the R STG-R culmen connection. Subsequently comparison between RSFC at R2 (subacute phase) and R1 revealed that majority (67%) of the ROI pairs, except four, had decreased connectivity. Increased connectivity of lingual, DMN, inferior-frontal, fronto-parietal networks were similar to the within subject contrast. Additionally, salience, DMN and precentral networks also had increased connections in this phase. All the ROIs that showed increased connections at R1 revealed decreased connections. One ROI pair, R Ang Gyr–R IPL remained unchanged between R1 and R2. All the four ROI pairs that showed increased connectivity in R2 had decreased connectivity at R1 and were interhemispheric (R SMG–L IFG, R SMG-L PCG, L MFG–R MFG). In the chronic phase (R2 vs. R3) all the ROI pairs except two (80%) had increased connectivity. The L POG–L STG, L Precun–L MFG that showed increased connectivity in the acute phase followed by reduction in the subacute phase did not show an increase in the chronic phase (R3).

#### Correlation between behavior scores and RSFC connectivity

**Figure 5:**
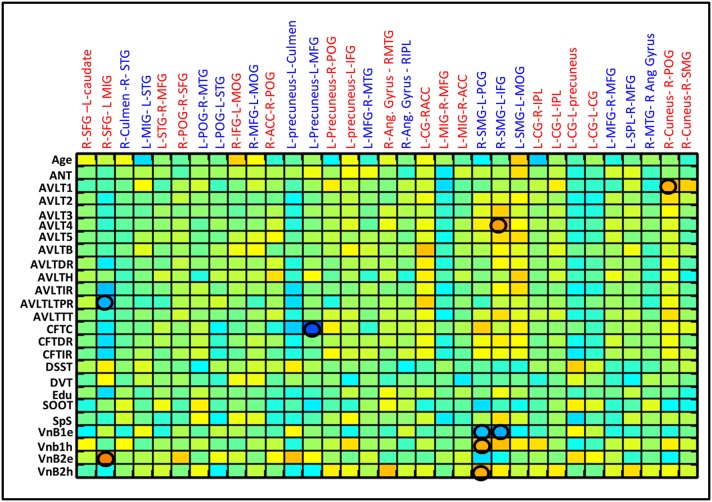
**Correlation between various behavior scores and the RSFC strength for the ROI pairs showing differences between CTRL and mild TBI as well as within mild TBI group**. Correlation matrices show the correlation between each possible pairs. Significant linear correlations are highlighted by a circle (*p* < 0.01).

All subjects at the initial evaluation within 12 days of injury had below normal scores for the various neuropsychological tests on attention, executive functions, learning, and memory. Sustained attention and sensory registration scores improved in first 3 months. Response speed, response inhibition, visuospatial memory, and visuospatial construction scores showed progressive recovery with maximum recovery in 1–3 month period and near normal recovery by 6 months post injury. Encoding and retrieval of learning and memory and category fluency improved overtime with a steep increase during the 3–6 months period. Linear correlation of behavioral scores with RSFC strength revealed five networks significantly correlated with the memory scores (Figure 5). The Lingual-DMN network connections correlated positively with the Auditory verbal learning test (AVLT) 1 scores and Posterior DMN-inferior frontal connectivity correlated negatively with complex figure tracing test. The connections of the salience network with DMN and precentral network correlated negatively with the Verbal n Back (VnB) errors. Salience-Pre central network connectivity positively correlated with VnB hits. Positive correlations of salience-DMN network were seen with AVLT4. Insula-precentral network were correlated positively with VnB errors and negatively with AVLT long-term potential retention.

## Discussion

We used resting state fMRI to define time varying connectivity changes in 15 patients as they recovered from uncomplicated mTBI using longitudinal resting functional connectivity analysis. Salient features of this study were that we recruited patients within 36 h of injury and excluded patients with potential PCS to define normal recovery. None of the patients had confounds of alcohol, drugs, prior trauma, neuropsychiatric or litigation issues. Thus, this was a very homogenous, uncomplicated group of patients with mTBI. We quantified the recovery from mTBI using both within mTBI group comparisons and comparing mTBI subjects at various time points in recovery to the group of healthy controls. Both types of analysis revealed that patients who recover within 6 months have generalized decreased brain connectivity in the first 3 months, with hyper connectivity of salience network, DMN and Posterior DMN within the first 36 h of injury. We also found that this hyper connectivity gradually spread and involved almost all the 15 networks and made them comparable to healthy controls 6 months after injury as the patient recovered in their neuropsychological scores.

Cross sectional studies in mTBI in the acute phase has revealed differential connectivity of several brain regions as reported by us with decreased connectivity of DMN and increased connectivity of rACC and ventrolateral prefrontal cortex (Mayer et al., 2011; Zhou et al., 2012) decreased motor striatal and increased fronto-parietal network connectivity (Shumskaya et al., 2012) and differential connectivity of DMN, hippocampus and thalamus (Iraji et al., 2015). The only other longitudinal study in mTBI involving 27 patients at 11 h and 3 months did not find any significant differences between the two time points (Mayer et al., 2011) although this study used a similar data analysis technique. Our results are in partial agreement with this as we also observed the changes to be most prominent between 3 and 6 months and not between 0 and 3 months (Figure 2). We found maximum changes in the contrasts linked with R3 in the analysis between R1, R2, and R3. In the second analysis comparing with HC it was noted that patients were significantly different at R1 and R2 and not at R3 suggesting that connectivity changes occurred between 3 and 6 months which made them comparable to HC at R3. Our findings of connectivity changes that varied with time is in tune with some of the principles of brain plasticity which found the complex environment induced plasticity of rats to be time dependent based on dendritic changes in the medial prefrontal and parietal cortex. There was evidence of increased spine density of the dendrites at 4 days in the medial frontal cortex, which reduced at 14 days when the parietal cortices started revealing increased spine density suggesting that plasticity induced by a novel environment varied with time and also that different regions responded differently with time (Comeau et al., 2010). Multiphasic nature of behavior recovery is well-established after mTBI (Brewer et al., 2002; Heitger et al., 2006; Tellier et al., 2009) with partial improvements in attention and executive functions within 3 months, and further improvements between 3 and 6 months (Heitger et al., 2006). Pattern of behavior recovery in our patients were also multiphasic and exploratory correlation with behavior score found linear correlation between behavior recovery and some of the network connectivity. Our finding of increased DMN and salience network connectivity within 36 h and reductions at 3 months, when lingual, DMN, inferior frontal and fronto-parietal networks revealed hyper connectivity is similar to the observations from experimental and behavioral studies and thus there is enough reason to believe that brain connectivity after injuries could also vary with time. Hence, the timing of imaging after injury is an important factor to be considered while we compare studies. This can partially explain the variability of results in the reported cross sectional literature, as time of recruitment after injury varied significantly in these cross sectional studies. Other reasons for variability—apart from variability induced by analysis methods and choice of ROIs could also include factors that can induce brain plasticity like sensory and motor experiences, task learning, hormones, drugs, aging, and stress as elaborated by Kolb and Muhammad (2014).

Fronto-parietal hyper connectivity in mTBI has already been documented in several studies (Mayer et al., 2011; Shumskaya et al., 2012) and was attributed to heightened environmental awareness and post concussive symptoms. With reports of hyper connectivity as a mechanism to unify various brain insults like TBI, Multiple sclerosis, Mild cognitive impairment, Alzheimers disease (Hillary et al., 2014a,b) and frequency of seizures in epilepsy (Bharath et al., 2015b), we are inclined to believe that these changes represent a response phenomenon of which the cause and mechanism of action is presently unknown. Our observation that these changes are spatially dynamic in the acute and sub-acute phase which spreads all over the brain at 6 months supports the hypothesis by Hillary et al., seeing hyper connectivity as a norm when resources are available. Since the hyper connectivity was most evident at 6 months when all patients had no significant PCS, it is unlikely be related to PCS and might be a normal response as patients recovered from a mTBI.

Unlike animal studies, factors affecting brain plasticity cannot be entirely controlled in humans and brain injuries are also extremely heterogeneous, so it is likely that there will be differences in detail between studies. We expect, however, the principle of time varying recovery to remain unaltered. For that same reason, we feel that the technical limitation of 0.25 mm difference in slice thickness between R1 and R2, R3, which was not regressed, would not have significantly affected our results. We also expect differences with varying time points of evaluation, as experimental evidence suggest that the large scale changes can be observed in minutes to hours following trauma. It is also possible that these changes can extend beyond the 6 month time point that we have chosen. The regions identified by both types of analysis were not the same as expected since the baselines were different and the estimation of significant differences were limited by the ROIs evaluated, (which did not include any regions in the cerebellum). Statistical significance was kept as *P* < 0.005 without correcting for multiple comparisons due to the sample size. Future studies using graph theory analysis could help in further characterizing whether the hyper connectivity has a non-random topology and also could evaluate the usefulness of neurocognitive therapy in the recovery. Despite these limitations and a modest sample size of 15 patients, our study remains important in understanding the natural course of recovery considering the difficulties associated with following up patients for 6 months after a mild head injury.

## Conclusion

This study in 15 patients with mild TBI reveals significant variations in resting state functional connectivity during the recovery window (36 h to 6 months) with the majority of the changes seen between 3 and 6 months after injury. Hyper connectivity of the salience and DMN networks within 36 h relaying to lingual, inferior frontal and fronto-parietal network at 3 months, and involving all 15 networks at 6 month demonstrates the time varying connectivity changes as the brain recovers from injury. It remains to be seen in future studies whether this acute phase hyper connectivity can be a potential predictor for an early recovery.

## Author contributions

RB, AM and IB contributed to the design of the work. AM, RP, JS, JR and DS contributed to the data acquisition and critically revised the manuscript. RB and SG contributed to data analysis, interpretation of the data, and drafting the manuscript. BB contributed to data analysis, interpretation, drafting the manuscript and critically revised the manuscript. All authors are accountable for all aspects of the work and approved the final version of the manuscript to be published.

## Conflict of interest statement

The authors declare that the research was conducted in the absence of any commercial or financial relationships that could be construed as a potential conflict of interest.
